# Supplementary material for: Adolescent Menstrual Health Literacy in Low, Middle and High-Income Countries: A Narrative Review
Source: Int J Environ Res Public Health. 2021 Feb 25;18(5):2260. doi: 10.3390/ijerph18052260 (PMC7956698; doi:10.3390/ijerph18052260)
Supplement: Supplementary file 1 [file ijerph-18-02260-s001.pdf]

Study	Study Design	Country[1]	Age Range	Sample Size	Population	Findings	Themes[2]
Abdelmoty et al (2015)	Cross-sectional questionnaire based study	Egypt (MIC)	11- 19	800	Female primary and secondary-school students enrolled in public school	"Four hundred twelve (51.5 %) out of 800 adolescents completed the questionnaire. 382 respondents reported various menstrual disorders, giving a prevalence rate of 95 %. Dysmenorrhea was the most prevalent (93 %) menstrual disorder in our sample, followed by PMS (65 %), and abnormal cycle lengths (43 %). Menstrual disorders interfered with social and academic life of 33 and 7.7 % of respondents respectively. Most participants lacked menstrual health knowledge and only 8.9 % of girls reported consulting a physician."	2
Adinma & Adinma (2008)	Cross- sectional descriptive study	Nigeria (MIC)	15 - 17	550	Secondary school girls in Co-educational & homogeneous girls' schools	"Abdominal pain/discomfort, (66.2%), was the commonest medical problem encountered by the respondents, although 45.8% had multiple problems. Analgesics, (75.6%), were most commonly used to relieve menstrual pain. Only 10% of respondents used non-pharmacologic remedies. Unsanitary menstrual absorbents were used by 55.7% of the respondents. Menstruation perceptions are poor, and practices often incorrect."	1,2,3
Agarwal & Venkat (2009)	Cross-sectional questionnaire based study	Singapore<br><br>(HIC)	12 - 19	5561	Girls from secondary schools and junior colleges	"Of the 5561 participants, 23.1% reported having irregular cycles. Oligomenorrhea was the most frequently reported problem (15.3%), and polymenorrhea was much less prevalent (2.0%). Dysmenorrhea was a significant problem, with 83.2% respondents reporting it in various degrees and 24% girls reporting school absenteeism owing to it. Dysmenorrhea was severe enough to require analgesics for pain relief in 45.1% of all subjects. In spite of menstrual problems being common, only 5.9% girls reported seeking medical advice for them."	2

[1] LIC = Low Income Country; MIC = Middle Income Country; HIC= High Income Country

[2] 1= Menstrual Hygiene Management; 2= Menstrual Health Issues; 3= Attitudes towards Menstruation

Ajah et al (2015)	Cross-sectional questionnaire study	Nigeria (MIC)	13 - 19	482	Girls from two secondary schools	"The declining age at menarche, menstrual challenges, and poor reproductive health status of adolescent girls in this study have made menstrual issues critical aspects of adolescent health care, especially when appropriate sources of reproductive health information were not fully harnessed."	1,2
Al-Maltouq et al (2019)	Cross-sectional study	Kuwait (HIC)	16 - 21	763	Adolescent girls from high-schools	"The one-year prevalence of dysmenorrhea was found to be 85.6% (95%CI: 83.1–88.1%). Of the participants with dysmenorrhea, 26% visited a public or a private clinic for their pain and 4.1% were hospitalized for their menstrual pain. Furthermore, 58.2% of students with dysmenorrhea missed at least one school day and 13.9% missed at least one exam."	3
Alam et al (2017)	Cross-sectional study	Bangladesh (MIC)	Not provided	2332	Schoolgirls from 700 schools (50 rural & 50 urban clusters)	"Students who felt uncomfortable at school during menstruation and who believed menstrual problems interfere with school performance were more likely to miss school during menstruation than those who did not. School absence during menstruation was less common among girls attending schools with unlocked toilet for girls. School absence was more common among girls who were forbidden from any activities during menstruation"	1,3
Amatya et al (2018)	Survey based study	Nepal (LIC)	Not provided	107	Adolescent girls in 2 local schools (Accham – rural area)	"The majority of the girls (n = 77, 72%) practiced exile, or Chhaupadi, during their menstruation, including 3 (4%) exiled to traditional Chhau sheds, 63 (82%) to livestock sheds, and 11 (14%) to courtyards outside their home. The remaining girls (n = 30, 28%) stayed inside the house, yet practiced some form of menstrual taboos. Of the 77 observed living spaces where the girls stayed during exile, only 30% (n = 23) had a toilet facility. Three of the girls were physically abused; nine were bitten by a snake. Notably high proportions of the living spaces lacked ventilation/windows (n = 20, 26%), electricity (n = 29, 38%), toilets (n = 54, 70%) and a warm blanket and mattress for sleeping (n = 29, 38%).	3

Ambresin et al (2012)	Cross-sectional survey based study	Switzerland<br><br>(HIC)	16 - 20	7548	Adolescents (48.5% female) attending high school or vocational school	"Patients with Severe Dysmenorrhea not only show a different profile from their peers in terms of their mental health academic track and gynecological age, but they are also more dissatisfied with their body appearance. Clinicians should pay particular attention to patients with SD and offer them a global evaluation, bearing in mind what factors can be associated with SD"	2
Behera, Sivakami & Behera (2015)	Qualitative research approach	India (MIC)	13 - 16	32	Adolescent girls in a rural secondary school	"Most of the participants, especially girls without menarche lacked adequate knowledge about menstruation and its processes. All communications regarding menstruation were between friends, whereas mothers were found to be playing a limited role. Some of the girls were using sanitary pads, while most girls still use old cloths. Financial concerns and difficulty in obtaining sanitary pads were major obstructions for their use. Lack of awareness and appropriate care for menstrual morbidities were reported among these girls, which put impact on their educational attainment."	1, 2
Blake et al (2018)	Intervention-based study	Ethiopia (LIC)	10 - 19	636	Adolescent girls from 10 intervention and 10 control schools in two rural districts	"The findings indicate that the distribution of *Growth and Changes* (or other similarly locally developed puberty books) may have an important role to play in equipping girls with the skills to navigate conversations with friends and close female relatives around a topic that is normally embarrassing or shameful to discuss. "	1, 3
Boden et al (2013)	Quantitative study	Sweden (HIC)	18 - 26	23	Endometriosis symptoms experienced by women during schooling	"Young women were found to be absent from school or missing extra-curricular activities due to pain and abnormal bleeding associated with their menstrual cycles. Some of the women thought that these symptoms were normal and did not consult the school nurse as a result of their limited experience. Others did not consult with the nurses because of a perception of poor treatment."	2,3
Boosey, Prestwich & Deave (2014)	Cross- sectional study	Uganda (LIC)	13 - 16	140	Girls from rural, government-run primary schools	"The girls reported a lack of access to adequate resources, facilities and accurate information to manage their menstrual hygiene effectively at school. They reported that, as a result, during menstruation they often struggle at school or miss school. "	1,2,3

Bush et al (2017)	Audit based study	New Zealand<br><br>(HIC)	13 - 18	2643	Male and Female students in co-ed schools and females in single sex schools, in public and private secondary schools	"In a region of consistent delivery of the education program, student awareness of endometriosis was 32% in 2015. Overall in 2015, 13% of students experienced distressing menstrual symptoms and 27% of students sometimes or always missed school due to menstrual symptoms. "	2
Chan et al (2009)	Questionnaire based study	China (MIC)	Not<br><br>Provide<br><br>d	5609	Girls from ten secondary schools<br>in Hong Kong	"The prevalence of menstrual problems in Hong Kong Chinese girls is high and causes significant disruption to their school and daily activities. However, only a minority seek medical advice."	2,3
Chang & Chen (2008)	Questionnaire based study	Taiwan (HIC)	10 - 12	417	Female students in 12 elementary schools in Hualien	"Elementary level female students who had better menarche preparation scored higher on the menstrual knowledge questionnaire, and those whose first period had already occurred felt more confident in their ability to execute proper menstrual health care behavior. The results prompted the recommendation to families and elementary schools to encourage students to improve their menstrual health care behavior during menses."	2,3

Chang et al (2009)	Qualitative research design	Taiwan (HIC)	10 - 12	20	Female elementary students	"Our findings show that young females can experience significant physical and emotional difficulties around menstruation – many of which stem from poor information and the reactions of their peer group to menstrual activity. "	2,3
Chang, Hayter & Lin (2011)	Qualitative study	Taiwan (HIC)	10 - 12	27	Male elementary students in Hualien	"Young boys have misguided knowledge about menstruation and this helps to perpetuate the stigma surrounding this element of reproductive health. Boys also express a desire to learn more but are often restricted in this by home and school. School nurses are the best placed professionals to address this issue."	3
Chinyama et al (2019)	Qualitative exploratory study	Zambia (MIC)	14 – 18 (student age only)	Not provided	Adolescent girls, boys, teachers, female guardians and traditional leaders from 6 rural schools	"Most girls reported learning about menstruation only at menarche and did not know the physiological basis of menstruation. They reported MHM-related challenges, including: use of non-absorbent and uncomfortable menstrual cloth and inadequate provision of sanitary materials, water, hygiene and sanitation facilities (WASH) in schools. In particular, toilets did not have soap and water or doors and locks for privacy and had a bad odor. Girls' school attendance and participation in physical activities was compromised when menstruating due to fear of teasing (especially by boys) and embarrassment from menstrual leakage. "	1, 3
Chothe et al (2014)	Qualitative study	India (MIC)	9 - 13	612	Three girls' schools	"Students had substantial doubts about menstruation and were influenced by societal myths and taboos in relation to menstrual practices"	1,2,3
Connolly & Sommer (2013)	Comparative case study	Cambodia (MIC)	Not provided	146	Girls from rural and urban schools	Key findings included girls' recommendations for teaching methodologies that encourage questions and practical content regarding puberty and menstrual management before the onset of menarche, and WASH-specific recommendations for the increased availability of water and sanitary materials in toilet stalls and greater privacy from boys and other girls"	1,2,3

Davis et al (2018)	Cross- sectional questionnaire based study	Indonesia<br><br>(MIC)	12 <	1159	Adolescent girls (50% from rural area) from 16 schools	"Over half reported poor MHM practices, and 11.1% had missed one or more days of school during their most recent menstrual period. Poor MHM practices were associated with rural residence, province, lower school grade and low knowledge of menstruation. High prevalence of poor MHM and considerable school absenteeism due to menstruation among Indonesian girls highlight the need for improved interventions.	1,2
Ellis et al (2016)	Qualitative data	Philippines<br><br>(MIC)	11 - 18	79	Adolescent girls from rural & metro schools	"Findings show that poor maintenance of facilities was one of the key WASH challenges identified by girls in managing menstruation in school. Poor access to water exacerbated other WASH conditions, impacting functionality and therefore student use of toilets. Girls are unable to manage menstruation at school when challenges with cleanliness, maintenance, and functionality of WASH facilities exist. These challenges at school are magnified by a lack of systems and oversight."	1
Fakhri et al (2012)	Quasi – experimental study	Iran (MIC)	14 - 18	698	Girls from selected high schools (rural & urban) in Mazandaran province	"Among the most significant results was the impact of educational sessions on bathing and genital hygiene. A total of 61.6% in the experimental group compared with 49.3% in the control group engaged in usual bathing during menstruation. Attitude towards menstruation was also significantly related to menstrual health."	1
Girod et al (2017)	Qualitative data	Kenya (MIC)	6 - 11	Not<br><br>provided	Adolescent girls from 6 primary schools	"Results showed that supply chains to public schools were not reliable, and equitable pad provision was not assured. Girls in private schools struggled to access pads because they were not provided. Sanitation facilities were physically available, but Muslim girls were unable to practice ablution due to the design of toilets in our study schools. Girls experienced fear and anxiety due to harassment from male peers and had incomplete information about menstruation from teachers."	1, 3

Gultie, Hailu & Workinyeh (2014)	Questionnaire based study	Ethiopia (LIC)	Not provided	492	Female students from Mehalmeda high school	"Four hundred forty six (90.7%) respondents had high level knowledge about menstrual hygiene management. Most of the respondents 457 (92.9%) and 475 (96.5%) had access for water and toilet facility respectively. "	1
Haque et al (2014)	Intervention Study	Bangladesh (MIC)	11- 16	416	Female adolescents from three schools	After health education, participants reported a significant improvement in their menstrual knowledge and beliefs.	1,2,3
Hennegan et al (2016)	Secondary analysis of survey data	Uganda (LIC)	10 - 19	205	Adolescent girls from rural primary schools	"90.5% of girls failed to meet available criteria for adequate MHM, with no significant difference between those using reusable sanitary and those using existing methods, predominantly cloth. Aspects of MHM predicted some consequences including shame, not standing in class to answer questions and concerns about odour"	1
Janoowalla (2020)	Interventional prospective cohort study	Rwanda (LIC)	18 - 24	209	Adolescent girls from four rural secondary schools	"The present study found no difference in the rate of UTI with and without the use of menstrual pads. A decreased risk of vulvovaginal symptoms in girls who used menstrual pads during the study was found. In addition, the knowledge, attitudes, and practices surrounding menstruation in a vulnerable and poorly studied population were able to be characterized. It was found that a significant percentage of participants had received little to no education or knowledge about menstruation before menarche. In addition, only one-third of participants used menstrual pads as their form of MHM."	1, 3

Jeon, Cha & Sok (2014)	Cross- sectional questionnaire study	Korea (HIC)	Not provided	572	Female students from 3 middle schools	"Nursing intervention programs for alleviating dysmenorrhea in Korean middle school adolescents are essential in order to reduce their level of stress, improve their perceived health status, and help them to maintain regular dietary habits. Reflecting on the recent trend of female students menstruating at a younger age, public health education courses and counseling programs should offer customized methods for alleviating dysmenorrhea."	2,3
Karkada et al (2012)	Community-based cross-sectional survey study	India (MIC)	12 - 15	166	Adolescent girls in selected rural high schools	" Out of 166 respondents, 54.2% girls were aware about menstruation prior to attainment of menarche. Mother was the first informant regarding menstruation in case of 4.8% girls. 27.7% girls believed it was a physiological process. Regarding practices, only 53.6% girls used sanitary pads during menstruation. Regarding restrictions practiced, 88% girls practiced different restrictions during menstruation."	1
Kazama, Maruyama & Nakamura (2015)	Cross- sectional questionnaire based study	Japan (HIC)	12 - 15	1018	Female junior high school students	"Dysmenorrhea that adversely affects daily activities is highly prevalent, and may be associated with certain lifestyle factors in junior high school students. Health education teachers should be made aware of these facts, and appropriately care for those suffering from dysmenorrhea symptoms, absentees, and those experiencing difficulties in school life due to dysmenorrhea symptoms."	2,3
Korir, Okwar & Okumbe (2018)	Cross- sectional questionnaire based study	Kenya (MIC)	Not provided	320	Adolescent girls from rural primary schools	"One third of pubescent school girls from the pastoralist community in Kajiado County, Kenya have sub-optimal MHM practices. The main contributors of this are lack of latrine privacy and fear of teasing by boys."	1, 3
Lahme, Stern & Cooper (2018)	Explorative study design	Zambia (MIC)	13 - 20	51	Females from three secondary schools (rural, urban and peri-urban)	"The paper shows that the girls suffer from poor menstrual hygiene, originating from lack of knowledge, culture and tradition, and socio-economic and environmental constraints, leading to inconveniences, humiliation and stress. This leads to reduced school attendance and poor academic performance, or even drop outs, and ultimately infringes upon the girls' human rights."	1,3

Mahajan & Kaushal (2017)	Cross- sectional descriptive study	India (MIC)	Not provided	100	Adolescent girls from government girls' schools	"The data on knowledge scores revealed that 29% had adequate knowledge about menstrual hygiene, 71% had inadequate knowledge about menstrual hygiene. The data revealed on practice scores revealed that 19%, 69%, 12% samples had poor, fair and good score of practices regarding menstrual hygiene respectively. "	1,3
Mason et al (2013)	Qualitative data analysis	Kenya (MIC)	14 - 16	120	Adolescent girls from 6 rural schools	"Emergent themes were: lack of preparation for menarche; maturation and sexual vulnerability; menstruation as an illness; secrecy, fear and shame of leaking; coping with inadequate alternatives; paying for pads with sex; and problems with menstrual hygiene. Parental and school support of menstrual needs is limited, and information sparse or inaccurate. Girls admitted 'others' rather than themselves were absent from school during menstruation, due to physical symptoms or inadequate sanitary protection. They described difficulties engaging in class, due to fear of smelling and leakage, and subsequent teasing. "	1, 2, 3
Mason et al (2017)	Qualitative research study	India (MIC)	13 - 17	85	Adolescent boys from 8 schools	"Some boys learnt about puberty and menstruation as part of the curriculum but had concerns this was not in-depth, or was missed out altogether. Most gathered knowledge from informal sources, from overhearing conversations or observing cultural rituals. Few boys openly displayed a negative attitude, although a minority voiced the idea that menstruation is a '*disease*'. Boys were mostly sympathetic to their menstruating sisters and wanted to support them."	3
McMahon et al (2011)	Qualitative data analysis	Kenya (MIC)	12 - 16	48 (girls) + 9	Adolescent girls from 6 rural schools	"Girls expressed fear, shame, distraction and confusion as feelings associated with menstruation. These feelings are largely linked to a sense of embarrassment, concerns about being stigmatized by fellow students and, as teachers explained, a perception that the onset of menstruation signals the advent of a girl's sexual status. Among the many methods for managing their periods, girls most frequently said they folded, bunched up or sewed cloth, including cloth from shirts or dresses, scraps of old cloth, or strips of an old blanket"	1, 3

				(teacher s)			
Miiro et al (2018)	Mixed methods research	Uganda (LIC)	14 - 17	352	Boys & girls from secondary schools	"Girls reported substantial embarrassment and fear of teasing related to menstruation in the qualitative interviews, and said that this, together with menstrual pain and lack of effective materials for menstrual hygiene management, led to school absenteeism"	1,2,3
Montgomery et al (2016)	Quasi-randomised controlled study	Uganda (LIC)	Not provided	1124	Eight schools in rural Uganda	"Results of the trial support the hypothesised positive impact of providing sanitary pads or puberty education for girls' school attendance in a developing country context. Findings must be interpreted with caution in light of poor participant retention, intervention fidelity, and the attendance measures"	1
Morrison et al (2018)	Parallel mixed method design	Nepal (LIC)	Not provided	860	Adolescent girls from six rural schools, one urban school, and one peri-urban school per district	"Girls in all districts experienced social, material and information barriers to confident menstrual management. Menstrual blood was believed to carry diseases, and girls' movement was restricted to contain ritual pollution and protect them from illness, spirit possession, and sexual experiences. Taboos prevented girls from worshipping in temples or in their home, and some girls were not allowed to enter the kitchen, or sleep in their home while menstruating. Teachers and parents felt unprepared to answer questions about menstruation and focused on the maintenance of restrictions."	1, 3
Mumtaz at el (2019)	Qualitative data	Pakistan (MIC)	16 - 19	312	Adolescent girls from six rural and urban school sites	"Three key themes emerged from our data: (1) a poorly maintained, girls-unfriendly School WASH infrastructure was a result of gender-insensitive design, a cultural devaluation of toilet cleaners and inadequate governing practices; (2) the design of WASH facilities did not align with traditionally-determined modes of disposal of rag-pads, the most common used	1, 3

						absorbents; (3) traditional menstrual management practices situate girls in an 'alternate space' characterised by withdrawal from many daily routines. These three socio-culturally determined practices interacted in a complex manner, often leading to interrupted class engagement and attendance."	
Muthuswamy et al (2019)	Survey based study	India (MIC)	>12	3617	Adolescent girls from 43 randomly selected schools across 3 states	"Menstrual hygiene education, accessible sanitary products, pain relief, and adequate sanitary facilities at school would improve the schooling-experience of adolescent girls in India."	1,2
Naeem, Klawitter & Aziz (2015)	Intervention based study	Pakistan (MIC)	Not provided	Not provided	Adolescent girls from 6 government high schools	"After the interventions data collection and analysis showed that the girls felt the interventions were helpful for them in dealing with MHM problems. A few of the girls felt that the general cleanliness of the school had improved; toilets and water tanks were cleaner than before the interventions."	1,2,3
Ortiz et al (2009)	Questionnaire based study	Mexico (MIC)	17 - 26	1152	Female high-school students	"Dysmenorrhea had a prevalence of 48.4% and was the cause of school absences for 24% of the affected students. It was mild in 32.9%, moderate in 49.7%, and severe in 17.4% of these students, of whom 28% consulted a physician and 60.9% self-medicated."	2
Parker, Sneddon & Arbon (2010)	Quantitative survey based study	Australia (HIC)	15 - 19	1051	Females from four senior high schools in Australian Capital Territory (state)	"Highly significant associations were found between increasing severity of menstrual pain, number of menstrual-related symptoms, interference with life activities and school absence. These associations indicate that approximately 25% of the sample had marked menstrual disturbance: 21% experienced severe pain; 26% school absence. Diagnosis of menstrual pathology in the sample was low, even though 33% had seen a GP and 9% had been referred to a specialist."	2

Pitangui et al (2013)	Cross- sectional descriptive study	Brazil (MIC)	12- 17	218	Females from public school in Petrolina, Brazil	"Dysmenorrhea had a prevalence of 73%, and school absenteeism was observed among 31% of the adolescents. In addition, 66% of the participants considered that dysmenorrhea affected their activities of daily living. Associations were found between the intensity of pain and the variables: school absenteeism; affected activities of daily living; need to use medications; and between affected activities of daily living and school absenteeism"	2
Rastogi, Khanna & Mathur (2019)	Cross-sectional study	India (MIC)	13 - 15	187	Adolescent girls from 4 govt. schools in Delhi	"Only 40% of girls were aware of menstruation prior to menarche. The majority of girls (95.7%) did not know the source of menstrual blood. About two-thirds (65.8%) of girls were unaware that the menstrual blood flow could be affected by anaemia. Approximately 17% of the girls believed a woman to be impure during menstruation. About half of the girls absented themselves from school for the first 2–3 days of their menstrual cycle. Thirty-four percent girls did not bathe at all during their menstrual period and a similar number of girls did not bathe daily while menstruating. Dysmenorrhoea was a common problem among 60% of girls. "	1, 2, 3
Rheinlander et al (2019)	Qualitative research approach	Ghana (MIC)	15 - 23	33	Adolescent girls in schools	"The study shows that senior school girls from a hygiene and sanitation deprived peri-urban community in Ghana are experiencing serious hygiene and menstrual poverty – on infrastructural, social and emotional levels. It is particularly concerning that girls have to struggle with negative moralizing and punishing Menstrual Hygiene Management discourses, thus resorting to secretive coping strategies."	1, 3
Sandhya et al (2013)	Intervention Study	Nepal (LIC)	15 - 22	350	Adolescent girls in a secondary rural school	"In the pre test 18.33% girls had good knowledge about menstrual hygiene, 60.0% had fair knowledge and 21.67% of them had poor knowledge on menstrual hygiene but in post test 43.33% had good knowledge on menstrual hygiene, 48.33% had fair knowledge and 8.34% of them had poor knowledge on the topic"	1,2,3

Santina, Wehbe & Ziade (2012)	Cross- sectional Questionnaire study	Lebanon (MIC)	13- 19	389	Adolescent girls from 5 English education high schools	"Significant predictors of dysmenorrhoea were negative menstrual experience, younger age of girl, longer duration of bleeding and longer cycle length. Better reproductive health educational programmes focusing on menstruation are needed for female adolescents."	2
Srivastava & Chandra (2017)	Questionnaire based study	India (MIC)	12 - 21	537	Girls from 5 high schools	"The study showed that most of the girls gained information about menstruation from their mothers. Menstruation is still considered as something shameful and young girls face many restrictions. Knowledge regarding menstruation, reproduction, contraception and diet is still lacking among the school girls. Family life skill sessions address these issues very effectively. Most of the girls enjoyed the sessions and were ready for more."	1,3
Su & Lindell (2016)	A quasi-experimental, pretest-posttest design	China (MIC)	12 - 15	116	Adolescent girls from 2 middle schools	"A nurse-managed education program improved adolescent girls' menstrual knowledge, promoted a more positive attitude, encouraged confidence, and improved pain relief practice. "	1,2,3
Suvitie et al (2016)	Cross- sectional study	Finland (HIC)	15 - 19	2582	Adolescent girls attending elementary school, high school, or vocational institute at 3 cities in Southwest Finland	"One-third (355/1089) of 15- to 19-year-old girls had severe menstrual pain and 14% (49/355) of them were regularly absent from school or hobbies. Five percent of all teenage girls (53/1103) were poor responders to conventional therapy for primary dysmenorrhea."	2

Tegegne & Sisay (2014)	Mixed-method research	Ethiopia (LIC)	10 - 19	595	Adolescent school girls	"Though there is an effort to increase girls' school enrolment, lack of basic needs, like sanitary napkins that facilitate routine activates of girls at early adolescence are observed to deter girls' school-attendance in rural Ethiopia."	1,3
Trinies et al (2015)	Qualitative interview-based study	Mali (LIC)	12- 17 (student age only)	26(girls) + 14 (others)	Adolescent girls, teachers & key informants from 8 urban & rural schools	"Girls lack information and school infrastructure is insufficient to match girls' personal preferences and the requirements of their practices and beliefs. The specifics within these areas have important implications for strategies to improve girls' experience managing menstrual hygiene at school"	1, 3
Um, Yusuf & Musa (2010)	Quantitative study	Nigeria (MIC)	10 - 19	400	Adolescent female secondary school students	"Majority had fair knowledge of menstruation, although deficient in specific knowledge areas. Most of them used sanitary pads as absorbent during their last menses; changed menstrual dressings about 1-5 times per day; and three quarter increased the frequency of bathing."	1,3
Upashe, Tekelab & Mekonnen (2015)	Cross-sectional questionnaire study	Ethiopia (LIC)	Not provided	828	High school girl students from ten high schools in Nekemte town	"In this study, 504 (60.9 %) and 330 (39.9 %) respondents had good knowledge and practice of menstrual hygiene respectively. The findings of the study showed a significant positive association between good knowledge of menstruation and educational status of mothers, having radio/TV. Educational status of the mother and earning permanent pocket money from parents revealed significant positive association with good practice of menstrual hygiene.	1
Vashisht et al (2018)	Mixed method research of combined cross-sectional study and qualitative research	India (MIC)	12 - 18	600	Adolescent girls from six secondary government schools in Delhi	"Out of 600, 245 (40%) girls remained absent from school during their menstruation. School absenteeism was significantly associated with the type of absorbent used, lack of privacy at school, restrictions imposed on girls during menstruation, mother's education, and source of information on menstruation. Nearly 65% reported that it affected their daily activities at school and that they had to miss their class tests and classes as a	1, 2, 3

						result of pain, anxiety, shame, anxiety about leakage, and staining of their uniform."	
Wijesiri et al (2012)	Descriptive study	Sri Lanka (MIC)	17 -18	200	Adolescent girls in a secondary school at Nugegoda Educational Division in the district of Colombo	" 84% of the study population had dysmenorrhea. Paracetamol was the drug of choice for pain relief. There was a statistically-significant ($P < 0.05$) association between pain and poor mental health status (66%) of the adolescent girls. Bathing was found to affect pain, as reported by 95% of the students. "	2
Wong (2011)	Focus group discussions	Malaysia (MIC)	13 - 19	172	Adolescent girls from 7 rural & urban public secondary schools	"Many participants revealed that they were not given or had not received detailed information about the mechanism or physiology of menstruation prior to its onset. Thus, many described the onset of menarche as shocking, an event for which they were unprepared, and which has had a tremendous impact on their emotions. More positive acceptance of menarche was reported in the urban than with the rural groups. "	2
Yadav et al (2018)	Cross- sectional questionnaire study	Nepal (LIC)	Not provided	276	Male & female adolescents from 11 rural schools	"67.4% respondents had fair knowledge and 26.4% respondents had good knowledge on menstrual hygiene management. However, out of 141 female adolescent respondents, only 56 (40%) were engaged in good menstrual hygiene practices. Around half of the respondents had positive attitude towards menstrual hygiene management related issues."	1,3
